# Supplementary material for: The Bidirectional Relationship between Chronic Kidney Disease and Hyperuricemia: Evidence from a Population-Based Prospective Cohort Study
Source: Int J Environ Res Public Health. 2023 Jan 18;20(3):1728. doi: 10.3390/ijerph20031728 (PMC9914133; doi:10.3390/ijerph20031728)
Supplement: Supplementary file 1 [file ijerph-20-01728-s001.zip › ijerph-2096844-supplementary.pdf]

Supplementary Material

The Bidirectional Relationship between Chronic Kidney Disease and Hyperuricemia: Evidence from a Population-Based Prospective Cohort Study

Zhibin Ma, Xiao Wang, Jia Zhang, Chao Yang, Hongmei Du, Feng Dou, Jianjian Li, Yini Zhao, Peiqin Quan and Xiaobin Hu *

Department of Epidemiology and Health Statistics, School of Public Health, Lanzhou University, Lanzhou 730000, China
* Correspondence: lzhuxb@126.com

Supplementary Table S1. Baseline characteristics of study participants in Analyses Ⅰ, stratified by CKD.
Supplementary Table S2. Baseline characteristics of study participants in Analyses II, stratified by HUA.
Supplementary Table S3. Baseline characteristics of study participants in Analyses III, stratified by gender.
Supplementary Table S4. Associations between dynamic change of eGFR with new-onset HUA.
Supplementary Table S5. Associations between dynamic change of sUA with new-onset CKD.
Supplementary Table S6. Associations between CKD and eGFR at baseline with new-onset HUA after excluding 352 participants who had outcomes during the first 2 years of follow-up (sensitivity analysis).
Supplementary Table S7. Associations between dynamic change of eGFR with new-onset HUA after excluding 352 participants who had outcomes during the first 2 years of follow-up (sensitivity analysis).
Supplementary Table S8. Associations between HUA and sUA at baseline with new-onset CKD after excluding 292 participants who experienced outcomes during the first 2 years of follow-up (sensitivity analysis).
Supplementary Table S9. Associations between dynamic change of sUA with new-onset HUA after excluding 292 participants who had outcomes during the first 2 years of follow-up (sensitivity analysis).
Supplementary Figure S1. Sub-analyses of restricted cubic spline of the association between eGFR with HUA, stratified by gender and age.
Supplementary Figure S2. Sub-analyses of restricted cubic spline of the association between sUA with CKD, stratified by gender and age.

Supplementary Figure S3. Sub-analyses of Cross-lagged model of the association between sUA with eGFR, stratified by gender and age (eGFR was estimated by the modified Modification of Diet in Renal Disease equation).

Supplementary Figure S4. Sub-analyses of the cross-lagged model of the association between sUA with eGFR, stratified by gender, age, and occupation, after excluding 195 participants with gout at baseline (sensitivity analysis).

Supplementary Table S1. Baseline characteristics of study participants in Analysis I, stratified by CKD

Variables	Total population (*N*=25,433)	CKD (*N*=749)	Non-CKD (*N*=24,684)	*p* Value
age (years), N (%)				<0.001
<45	13,121 (51.59)	291 (38.85)	12,830 (51.98)	
45~64	9783 (38.47)	286 (38.19)	9497 (38.47)	
≥65	2529 (9.94)	172 (22.96)	2357 (9.55)	
BMI (kg·m^{-2}), Mean ± SD	23.34 ± 3.13	24.62 ± 3.59	23.30 ± 3.11	<0.001
Male, N (%)	14,899 (58.58)	509 (67.96)	14,390 (58.30)	<0.001
Education, N (%)				<0.001
Junior high school or below	10,168 (39.98)	372 (49.67)	9796 (39.68)	
High school	6908 (27.16)	177 (23.63)	6731 (27.27)	
Junior college	4992 (19.63)	120 (16.02)	4872 (19.74)	
Bachelor's degree or above	3365 (13.23)	80 (10.68)	3285 (13.31)	
Occupation, N (%)				0.418
Front-line worker	19,599 (77.06)	568 (75.83)	19,031 (77.10)	
White-collar worker	5834 (22.94)	181 (24.17)	5653 (22.90)	
Smoking status, N (%)				<0.001
Non-smoker	14,389 (56.58)	370 (49.40)	14,019 (56.79)	
Smoker	8997 (35.37)	292 (38.99)	8705 (35.27)	
Ex-smoker	2047 (8.05)	87 (11.61)	1960 (7.94)	
Drinking status, N (%)				<0.001
Non-drinker	19,425 (76.38)	565 (75.43)	18,860 (76.41)	
Drinker	4904 (19.28)	130 (17.36)	4774 (19.34)	
Ex-drinker	1104 (4.34)	54 (7.21)	1050 (4.25)	
Physical exercise, N (%)				0.072
No	3418 (13.44)	115 (15.35)	3303 (13.38)	
Occasionally	9967 (39.19)	266 (35.52)	9701 (39.30)	
Often	12,048 (47.37)	368 (49.13)	11,680 (47.32)	
Diabetes, N (%)	1881 (7.40)	195 (26.03)	1686 (6.83)	<0.001
Hypertension, N (%)	7125 (28.01)	394 (52.60)	6731 (27.27)	<0.001
SBP (mmHg), Mean ± SD	122.76 ± 19.49	133.82 ± 24.87	122.43 ± 19.20	<0.001
DBP (mmHg), Mean ± SD	78.28 ± 12.12	84.30 ± 15.35	78.09 ± 11.96	<0.001
FPG (mmol/L), Mean ± SD	5.35 ± 1.46	6.57 ± 3.11	5.31 ± 1.36	<0.001
TC (mmol/L), Mean ± SD	4.67 ± 0.89	4.80 ± 1.00	4.67 ± 0.88	<0.001
TG (mmol/L), Mean ± SD	1.85 ± 1.44	2.25 ± 1.93	1.83 ± 1.42	<0.001
HDL-C (mmol/L), Mean ± SD	1.38 ± 0.35	1.32 ± 0.36	1.39 ± 0.35	<0.001
LDL-C (mmol/L), Mean ± SD	3.04 ± 0.74	3.11 ± 0.82	3.04 ± 0.74	0.011
sUA (μmol/L), Mean ± SD	303.86 ± 61.47	319.84 ± 58.30	303.37 ± 61.50	<0.001
eGFR (mL·min^{-1}·1.73m^{-2}), Mean ± SD	103.69 ± 13.80	92.76 ± 24.90	104.02 ± 13.18	<0.001

Data were presented as means ± SD for continuous variables and numbers (percentages) for categorical variables. Differences between groups were compared using the Student's t-test and Chi-squared test for categorical variables.

CKD, chronic kidney disease; HUA, hyperuricemia; SBP, systolic blood pressure; DBP, diastolic blood pressure; BMI, body mass index; FPG, fasting plasma glucose; TC, total cholesterol; TG, triglyceride; HDL-C, high-density lipoprotein cholesterol; LDL-C, low-

density lipoprotein cholesterol; sUA, serum uric acid; eGFR, estimated glomerular filtration rate.

Supplementary Table S2. Baseline characteristics of study participants in Analysis II, stratified by HUA

Variables	Total population (*N*=28,422)	HUA (*N*=3869)	Non-HUA (*N*=24,553)	*p* Value
age (years), N (%)				<0.001
<45	14,816 (52.13)	2023 (52.29)	12,793 (52.10)	
45~64	10,833 (38.11)	1407 (36.37)	9426 (38.39)	
≥65	2773 (9.76)	439 (11.34)	2334 (9.51)	
BMI (kg·m^{-2}), Mean ± SD	23.83 ± 3.02	24.73 ± 3.02	23.69 ± 3.00	<0.001
Male, N (%)	17,536 (61.70)	3189 (82.42)	14,347 (58.43)	<0.001
Education, N (%)				<0.001
Junior high school or below	11,103 (39.06)	1381 (35.69)	9722 (39.60)	
High school	7776 (27.36)	1077 (27.84)	6699 (27.28)	
Junior college	5669 (19.95)	812 (20.99)	4857 (19.78)	
Bachelor's degree or above	3874 (13.63)	599 (15.48)	3275 (13.34)	
Occupation, N (%)				0.193
Front-line worker	21,983 (77.35)	2961 (76.53)	19,022 (77.47)	
White-collar worker	6439 (22.65)	908 (23.47)	5531 (22.53)	
Smoking status, N (%)				<0.001
Non-smoker	15,459 (54.39)	1534 (39.65)	13,925 (56.71)	
Smoker	10,565 (37.17)	1889 (48.82)	8676 (35.34)	
Ex-smoker	2398 (8.44)	446 (11.53)	1952 (7.95)	
Drinking status, N (%)				<0.001
Non-drinker	21,128 (74.34)	2375 (61.39)	18,753 (76.38)	
Drinker	6022 (21.19)	1263 (32.64)	4759 (19.38)	
Ex-drinker	1272 (4.47)	231 (5.97)	1041 (4.24)	
Physical exercise, N (%)				0.008
No	3854 (13.56)	542 (14.01)	3312 (13.49)	
Occasionally	11,233 (39.52)	1601 (41.38)	9632 (39.23)	
Often	13,335 (46.92)	1726 (44.61)	11,609 (47.28)	
Diabetes, N (%)	1947 (6.85)	280 (7.24)	1667 (6.79)	0.306
Hypertension, N (%)	8286 (29.15)	1604 (41.46)	6682 (27.21)	<0.001
SBP (mmHg), Mean ± SD	123.35 ± 19.33	129.40 ± 19.05	122.40 ± 19.20	<0.001
DBP (mmHg), Mean ± SD	78.65 ± 12.17	82.19 ± 12.92	78.10 ± 11.95	<0.001
FPG (mmol/L), Mean ± SD	5.32 ± 1.32	5.40 ± 1.09	5.31 ± 1.35	<0.001
TC (mmol/L), Mean ± SD	4.70 ± 0.89	4.88 ± 0.94	4.67 ± 0.88	<0.001
TG (mmol/L), Mean ± SD	1.94 ± 1.52	2.62 ± 1.90	1.83 ± 1.42	<0.001
HDL-C (mmol/L), Mean ± SD	1.37 ± 0.35	1.25 ± 0.32	1.39 ± 0.35	<0.001
LDL-C (mmol/L), Mean ± SD	3.05 ± 0.74	3.14 ± 0.75	3.04 ± 0.73	<0.001
sUA (μmol/L), Mean ± SD	324.19 ± 79.52	455.77 ± 49.09	303.45 ± 61.50	<0.001
eGFR (mL·min^{-1}·1.73m^{-2}), Mean ± SD	103.51 ± 13.53	100.07 ± 15.14	104.05 ± 13.17	<0.001

Data were presented as means ± SD for continuous variables and numbers (percentages) for categorical variables. Differences between groups were compared using the Student's t-test and Chi-squared test for categorical variables.

CKD, chronic kidney disease; HUA, hyperuricemia; SBP, systolic blood pressure; DBP, diastolic blood pressure; BMI, body mass index; FPG, fasting plasma glucose; TC, total cholesterol; TG, triglyceride; HDL-C, high-density lipoprotein cholesterol; LDL-C, low-

density lipoprotein cholesterol; sUA, serum uric acid; eGFR, estimated glomerular filtration rate.

Supplementary Table S3. Baseline characteristics of study participants in Analysis III, stratified by gender

Variables	Total population (*N*=31,028)	Male (*N*=19,118)	Female (*N*=11,910)	*p* Value
age (years), N (%)				<0.001
<45	16,064 (51.77)	9973 (52.16)	6091 (51.14)	
45~64	11,759 (37.90)	6821 (35.68)	4938 (41.46)	
≥65	3205 (10.33)	2324 (12.16)	881 (7.40)	
BMI ($kg \cdot m^{-2}$), Mean ± SD	23.61 ± 3.22	24.09 ± 3.06	22.83 ± 3.30	<0.001
Education, N (%)				<0.001
Junior high school or below	12,238 (39.44)	7145 (37.37)	5093 (42.76)	
High school	8430 (27.17)	5421 (28.36)	3009 (25.26)	
Junior college	6163 (19.86)	3985 (20.84)	2178 (18.29)	
Bachelor's degree or above	4197 (13.53)	2567 (13.43)	1630 (13.69)	
Occupation, N (%)				<0.001
Front-line worker	23,919 (77.09)	15,362 (80.35)	8557 (71.85)	
White-collar worker	7109 (22.91)	3756 (19.65)	3353 (28.15)	
Smoking status, N (%)				<0.001
Non-smoker	16,878 (54.40)	5161 (26.99)	11,717 (98.38)	
Smoker	11,507 (37.08)	11,350 (59.37)	157 (1.32)	
Ex-smoker	2643 (8.52)	2607 (13.64)	36 (0.30)	
Drinking status, N (%)				<0.001
Non-drinker	23,046 (74.27)	11,462 (59.95)	11,584 (97.26)	
Drinker	6562 (21.15)	6272 (32.81)	290 (2.44)	
Ex-drinker	1420 (4.58)	1384 (7.24)	36 (0.30)	
Physical exercise, N (%)				<0.001
No	4232 (13.64)	2840 (14.85)	1392 (11.69)	
Occasionally	12,283 (39.59)	7612 (39.82)	4671 (39.22)	
Often	14,513 (46.77)	8666 (45.33)	5847 (49.09)	
Diabetes, N (%)	2321 (7.48)	1679 (8.78)	642 (5.39)	<0.001
Hypertension, N (%)	9356 (30.15)	6404 (33.50)	2952 (24.79)	<0.001
CKD, N (%)	1136 (3.66)	802 (4.19)	334 (2.80)	<0.001
HUA, N (%)	4539 (14.63)	3715 (19.43)	824 (6.92)	<0.001
SBP (mmHg), Mean ± SD	123.74 ± 19.66	126.27 ± 18.94	119.68 ± 20.10	<0.001
DBP (mmHg), Mean ± SD	78.87 ± 12.39	80.12 ± 12.47	76.86 ± 11.99	<0.001
FPG (mmol/L), Mean ± SD	5.35 ± 1.41	5.45 ± 1.52	5.20 ± 1.21	<0.001
TC (mmol/L), Mean ± SD	4.70 ± 0.90	4.66 ± 0.88	4.77 ± 0.92	<0.001
TG (mmol/L), Mean ± SD	1.96 ± 1.54	2.18 ± 1.70	1.60 ± 1.16	<0.001
HDL-C (mmol/L), Mean ± SD	1.36 ± 0.35	1.28 ± 0.32	1.50 ± 0.35	<0.001
LDL-C (mmol/L), Mean ± SD	3.06 ± 0.74	3.02 ± 0.73	3.11 ± 0.76	<0.001
sUA (μmol/L), Mean ± SD	326.41 ± 81.62	361.33 ± 73.88	270.36 ± 59.11	<0.001
eGFR ($mL \cdot min^{-1} \cdot 1.73m^{-2}$), Mean ± SD	102.94 ± 14.63	102.54 ± 14.70	103.57 ± 14.49	<0.001

Data were presented as means ± SD for continuous variables and numbers (percentages) for categorical variables. Differences between groups were compared using the Student's

t-test and Chi-squared test for categorical variables.
CKD, chronic kidney disease; HUA, hyperuricemia; SBP, systolic blood pressure; DBP, diastolic blood pressure; BMI, body mass index; FPG, fasting plasma glucose; TC, total cholesterol; TG, triglyceride; HDL-C, high-density lipoprotein cholesterol; LDL-C, low-density lipoprotein cholesterol; sUA, serum uric acid; eGFR, estimated glomerular filtration rate.

Supplementary Table S4. Associations between dynamic change of eGFR with new-onset HUA.

Groups	N	No of Events (%)	Model 1		Model 2	
			HR (95% *CI*)	*p* Value	*HR* (95% *CI*)	*p* Value
N-N	25,107	1506 (6.00%)	1.00		1.00	
N-R	191	56 (29.32%)	3.32 (2.54-4.34)	<0.001	3.12 (2.36-4.13)	<0.001
R-N	73	8 (10.96%)	1.83 (0.92-3.67)	0.087	1.65 (0.82-3.31)	0.161
R-R	62	27 (43.55%)	4.49 (3.07-6.57)	<0.001	3.90 (2.63-5.77)	<0.001

Model 1 was not adjusted for any covariates.

Model 2 was adjusted for age (<45 years, 45-64 years, ≥65 years), gender (male, female), BMI (<24.0 $kg \cdot m^{-2}$, 24.0-27.9 $kg \cdot m^{-2}$, ≥28 $kg \cdot m^{-2}$), education (junior high school or below, high school, junior college, bachelor's degree or above), smoking status (non-smoker, smoker, ex-smoker), drinking status (non-drinker, drinker, ex-drinker), hypertension (no, yes), TC (≤4.20 mmol/L, 4.21-2.00 mmol/L, ≥5.01 mmol/L) at baseline.

The N-N group referred to normal eGFR at baseline and follow-up, the N-R group referred to normal eGFR at baseline and reduced eGFR at follow-up, and the R-N referred to reduced eGFR at baseline and normal eGFR at follow-up. The R-R group referred to reduced eGFR at both baseline and follow-up, where the reduced eGFR was defined when it was less than 60 $mL \cdot min^{-1} \cdot 1.73m^{-2}$, otherwise normal.

eGFR, estimated glomerular filtration rate; HUA, hyperuricemia; *HR*, hazard ratio; *CI*, confidence interval.

Supplementary Table S5. Associations between dynamic change of sUA with new-onset CKD.

Groups	N	No of Events (%)	Model 1		Model 2	
			HR (95% *CI*)	*p* Value	*HR* (95% *CI*)	*p* Value
N-N	23,062	794 (3.44%)	1.00		1.00	
N-H	1491	139 (9.32%)	2.05 (1.71-2.46)	<0.001	1.80 (1.50-2.16)	<0.001
H-N	2037	99 (4.86%)	1.28 (1.04-1.58)	0.020	0.97 (0.78-1.20)	0.742
H-H	1832	180 (9.83%)	2.40 (2.04-2.82)	<0.001	1.83 (1.54-2.16)	<0.001

Model 1 was not adjusted for any covariates.

Model 2 was adjusted for age (<45 years, 45-64 years, ≥65 years), gender (male, female), BMI (<24.0 kg·m^{-2}, 24.0-27.9 kg·m^{-2}, ≥28 kg·m^{-2}), smoking status (non-smoker, smoker, ex-smoker), drinking status (non-drinker, drinker, ex-drinker), diabetes (no, yes), hypertension (no, yes), TG (≤1.20 mmol/L, 1.21-2.00 mmol/L, ≥2.01 mmol/L) at baseline.

The N-N group referred to normal sUA at baseline and follow-up, the N-H group referred to normal sUA at baseline and hyperuricemia at follow-up, and the H-N referred to hyperuricemia at baseline and normal sUA at follow-up. The H-H group referred to elevated sUA at both baseline and follow-up, where the hyperuricemia was defined as sUA > 420 μmol/L in males and sUA > 360 μmol/L in females, otherwise normal.

sUA, serum uric acid; CKD, chronic kidney disease; *HR*, hazard ratio; *CI*, confidence interval.

Supplementary Table S6. Associations between CKD and eGFR at baseline with new-onset HUA after excluding 352 participants who had outcomes during the first 2 years of follow-up (sensitivity analysis).

	N	No of Events (%)	Model 1		Model 2	
			HR (95% *CI*)	*p* Value	*HR* (95% *CI*)	*p* Value
CKD at Baseline						
Non-CKD	24,346	1164 (4.78%)	1.00		1.00	
CKD	735	81 (11.02%)	2.09 (1.67-2.62)	<0.001	1.66 (1.32-2.09)	<0.001
Groups of eGFR ($mL \cdot min^{-1} \cdot 1.73m^{-2}$)						
Group1 (<60)	134	34 (25.37%)	4.77 (3.38-6.73)	<0.001	3.72 (2.59-5.33)	<0.001
Group2 (60~<90)	3635	384 (10.56%)	1.88 (1.66-2.12)	<0.001	1.60 (1.39-1.84)	<0.001
Group3 (90~<120)	18,956	764 (4.03%)	1.00		1.00	
Group4 (≥120)	2356	63 (2.67%)	1.36 (1.05-1.76)	0.020	1.67 (1.26-2.21)	<0.001

Model 1 was not adjusted for any covariates.

Model 2 was adjusted for age (<45 years, 45-64 years, ≥65 years), gender (male, female), BMI (<24.0 $kg \cdot m^{-2}$, 24.0-27.9 $kg \cdot m^{-2}$, ≥28 $kg \cdot m^{-2}$), education (junior high school or below, high school, junior college, bachelor's degree or above), smoking status (non-smoker, smoker, ex-smoker), drinking status (non-drinker, drinker, ex-drinker), hypertension (no, yes), TC (≤4.20 mmol/L, 4.21-2.00 mmol/L, ≥5.01 mmol/L) at baseline.

CKD, chronic kidney disease; eGFR, estimated glomerular filtration rate; HUA, hyperuricemia; *HR*, hazard ratio; *CI*, confidence interval.

Supplementary Table S7. Associations between dynamic change of eGFR with new-onset HUA after excluding 352 participants who had outcomes during the first 2 years of follow-up (sensitivity analysis).

Groups	N	No of Events (%)	Model 1		Model 2	
			HR (95% *CI*)	*p* Value	*HR* (95% *CI*)	*p* Value
N-N	24,760	1159 (4.68%)	1.00		1.00	
N-R	187	52 (27.81%)	3.78 (2.86-4.99)	<0.001	3.14 (2.34-4.20)	<0.001
R-N	73	8 (10.96%)	2.43 (1.21-4.87)	0.012	1.91 (0.95-3.84)	0.070
R-R	61	26 (42.62%)	5.23 (3.54-7.71)	<0.001	3.87 (2.59-5.79)	<0.001

Model 1 was not adjusted for any covariates.

Model 2 was adjusted for age (<45 years, 45-64 years, ≥65 years), gender (male, female), BMI (<24.0 kg·m^{-2}, 24.0-27.9 kg·m^{-2}, ≥28 kg·m^{-2}), education (junior high school or below, high school, junior college, bachelor's degree or above), smoking status (non-smoker, smoker, ex-smoker), drinking status (non-drinker, drinker, ex-drinker), hypertension (no, yes), TC (≤4.20 mmol/L, 4.21-2.00 mmol/L, ≥5.01 mmol/L) at baseline.

The N-N group referred to normal eGFR at baseline and follow-up, the N-R group referred to normal eGFR at baseline and reduced eGFR at follow-up, and the R-N referred to reduced eGFR at baseline and normal eGFR at follow-up. The R-R group referred to reduced eGFR at both baseline and follow-up, where the reduced eGFR was defined when it was less than 60 mL·min^{-1}·1.73m^{-2}, otherwise normal.

eGFR, estimated glomerular filtration rate; HUA, hyperuricemia; *HR*, hazard ratio; *CI*, confidence interval.

Supplementary Table S8. Associations between HUA and sUA at baseline with new-onset CKD after excluding 292 participants who experienced outcomes during the first 2 years of follow-up (sensitivity analysis).

	N	No of Events (%)	Model 1		Model 2	
			***HR* (95% *CI*)**	***p* Value**	***HR* (95% *CI*)**	***p* Value**
HUA at Baseline						
Non-HUA	24,316	696 (2.86%)	1.00		1.00	
HUA	3814	224 (5.87%)	1.77 (1.53-2.06)	<0.001	1.40 (1.20-1.64)	<0.001
sUA (μmol/L)						
Q1 (≤285)	6969	137 (1.97%)	1.00		1.00	
Q2 (266-318)	7017	190 (2.71%)	1.21 (0.97-1.50)	0.092	1.06 (0.84-1.33)	0.621
Q3 (319-374)	7017	235 (3.35%)	1.50 (1.21-1.85)	<0.001	1.14 (0.91-1.44)	0.250
Q4 (≥375)	7127	358 (5.02%)	2.15 (1.77-2.62)	<0.001	1.24 (1.16-1.84)	0.001
P_{trend}			<0.001		<0.001	

Model 1 was not adjusted for any covariates.
Model 2 was adjusted for age (<45 years, 45-64 years, ≥65 years), gender (male, female), BMI (<24.0 kg·m^{-2}, 24.0-27.9 kg·m^{-2}, ≥28 kg·m^{-2}), smoking status (non-smoker, smoker, ex-smoker), drinking status (non-drinker, drinker, ex-drinker), diabetes (no, yes), hypertension (no, yes), TG (≤1.20 mmol/L, 1.21-2.00 mmol/L, ≥2.01 mmol/L) at baseline.
Q1-Q4 referred to the quartiles of serum uric acid grouped according to the quartiles of the non-CKD participants, respectively. The median of each group was included in the regression model as a continuous variable to calculate the P value for the test of trend. HUA, hyperuricemia; sUA, serum uric acid; SBP, systolic blood pressure; CKD, chronic kidney disease; *HR*, hazard ratio; *CI*, confidence interval.

Supplementary Table S9. Associations between dynamic change of sUA with new-onset HUA after excluding 292 participants who had outcomes during the first 2 years of follow-up (sensitivity analysis).

Groups	N	No of Events (%)	Model 1		Model 2	
			HR (95% *CI*)	*p* Value	*HR* (95% *CI*)	*p* Value
N-N	22,849	581 (2.54%)	1.00		1.00	
N-H	1467	115 (7.84%)	2.18 (1.79-2.67)	<0.001	1.84 (1.51-2.27)	<0.001
H-N	2019	81 (4.01%)	1.41 (1.11-1.77)	0.004	1.12 (0.89-1.42)	0.339
H-H	1795	143 (7.97%)	2.49 (2.07-2.99)	<0.001	1.92 (1.59-2.33)	<0.001

Model 1 was not adjusted for any covariates.

Model 2 was adjusted for age (<45 years, 45-64 years, ≥65 years), gender (male, female), BMI (<24.0 kg·m^{-2}, 24.0-27.9 kg·m^{-2}, ≥28 kg·m^{-2}), smoking status (non-smoker, smoker, ex-smoker), drinking status (non-drinker, drinker, ex-drinker), diabetes (no, yes), hypertension (no, yes), TG (≤1.20 mmol/L, 1.21-2.00 mmol/L, ≥2.01 mmol/L) at baseline.

The N-N group referred to normal sUA at baseline and follow-up, the N-H group referred to normal sUA at baseline and hyperuricemia at follow-up, and the H-N referred to hyperuricemia at baseline and normal sUA at follow-up. The H-H group referred to elevated sUA at both baseline and follow-up, where the hyperuricemia was defined as sUA > 420 μmol/L in males and sUA > 360 μmol/L in females, otherwise normal.

sUA, serum uric acid; CKD, chronic kidney disease; *HR*, hazard ratio; *CI*, confidence interval.

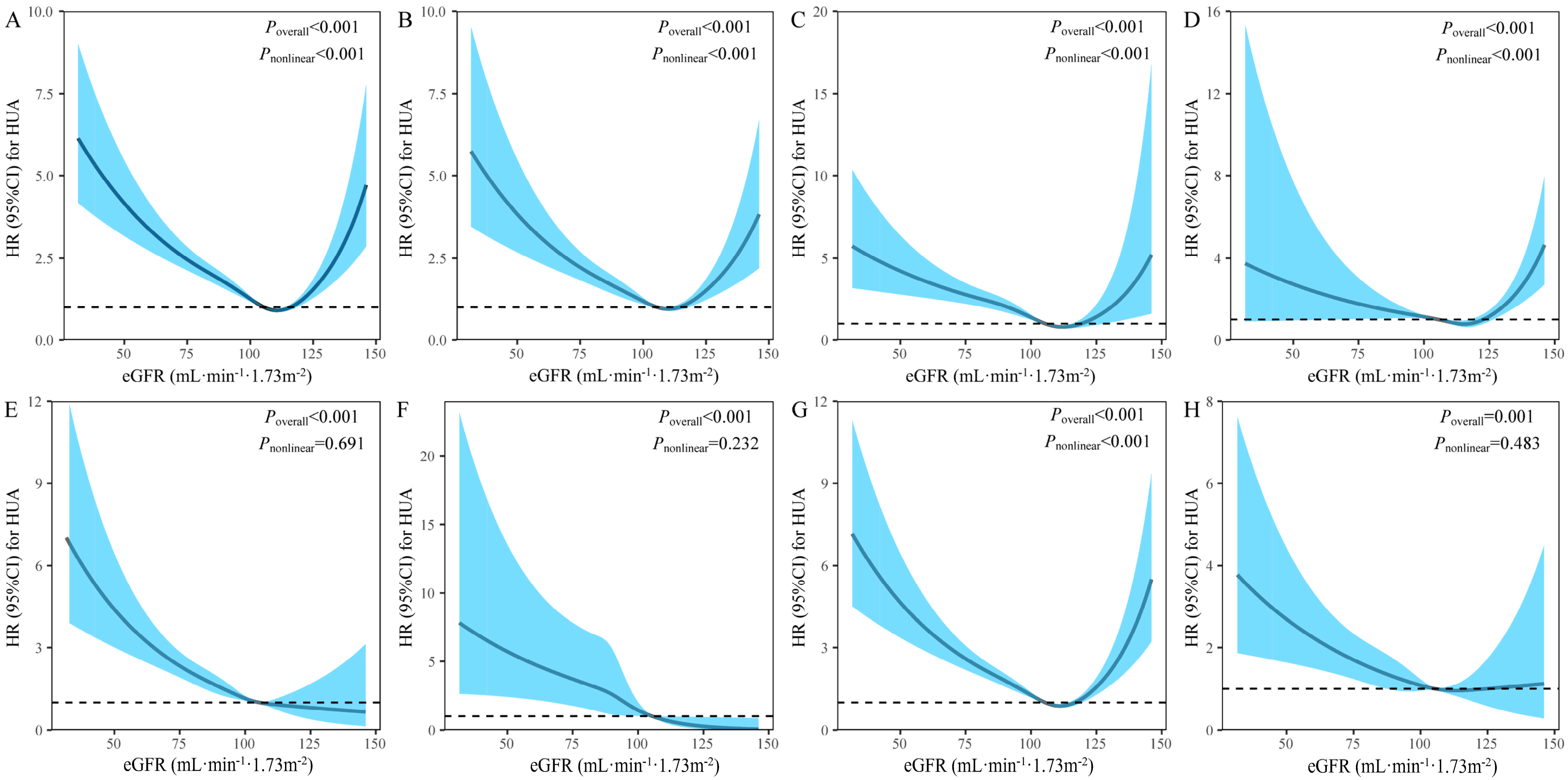

Supplementary Figure S1. Sub-analyses of the restricted cubic spline of the association between eGFR with HUA, stratified by gender, age, and occupation.

(A: Total population, B: male, C: female, D: age < 45 years, E: age 45-64 years, F: age ≥65 years, G: front-line workers, H: white-collar workers). The solid lines represented adjusted hazard ratios according to the change of eGFR, while the blue shading represented the 95% confidence intervals. Knots were placed at the 5th, 35th, 65th, and 95th percentiles, with the 50th percentiles set as a reference for eGFR. Covariates included in the model were age (< 45 years, 45-64 years, ≥ 65 years), gender (male, female), BMI (< 24.0 kg·m^{-2}, 24.0-27.9 kg·m^{-2}, ≥ 28 kg·m^{-2}), education (junior high school or below, high school, junior college, bachelor's degree or above), smoking status (non-smoker, smoker, ex-smoker), drinking status (non-drinker, drinker, ex-drinker), hypertension (no, yes), TC (≤4.20 mmol/L, 4.21-2.00 mmol/L, ≥5.01 mmol/L) at baseline when they were not the strata variables.
eGFR, estimated glomerular filtration rate; HUA, hyperuricemia; *HR*, hazard ratio; *CI*, confidence interval.

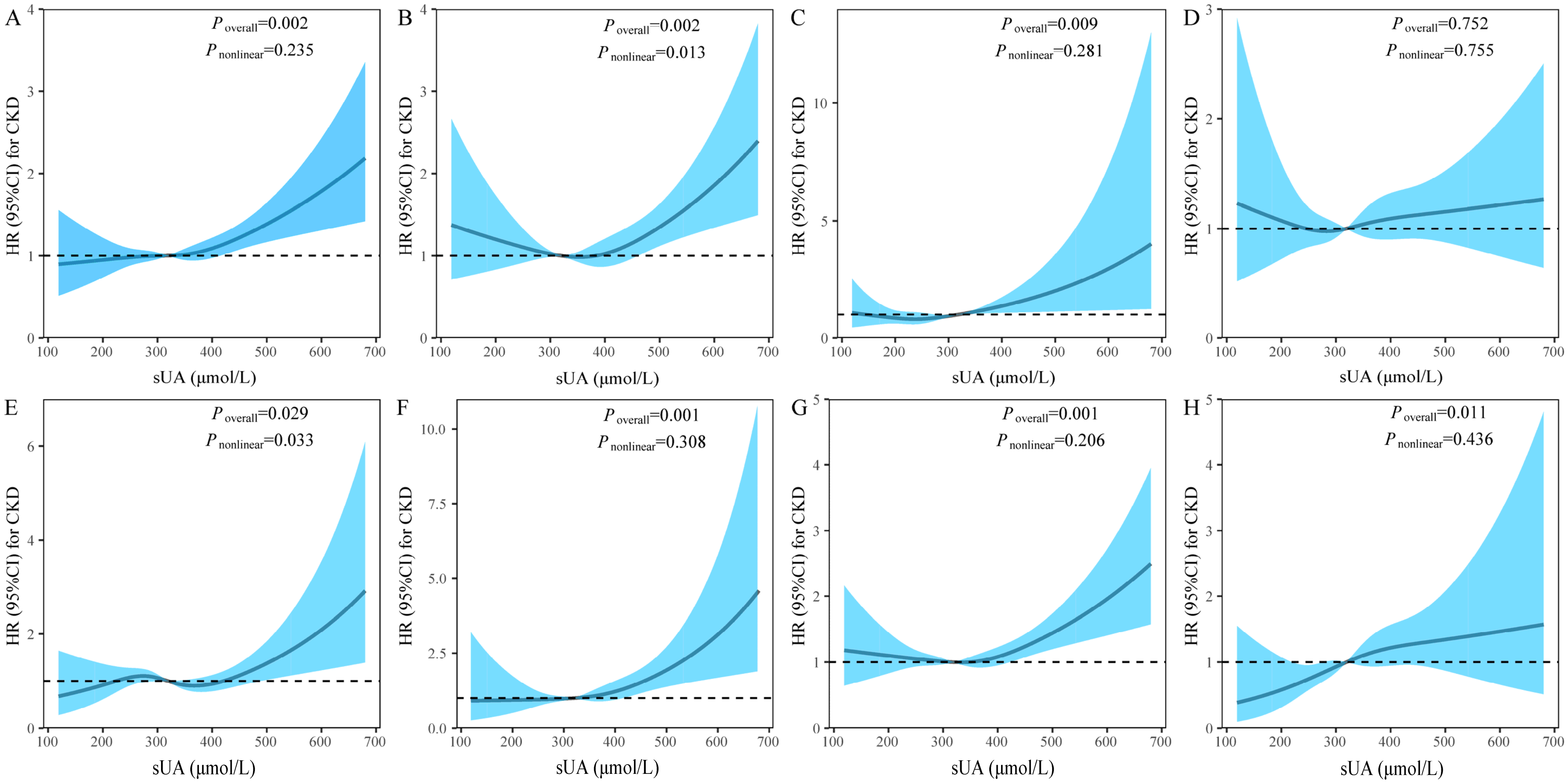

Supplementary Figure S2. Sub-analyses of the restricted cubic spline of the association between sUA with CKD, stratified by gender, age, and occupation.

(A: Total population, B: male, C: female, D: age < 45 years, E: age 45-64 years, F: age ≥65 years, G: front-line workers, H: white-collar workers). The

solid lines represented adjusted hazard ratios according to the change of sUA, while the blue shading represented the 95% confidence intervals. Knots were placed at the 5th, 35th, 65th, and 95th percentiles, with the 50th percentiles set as a reference for sUA and eGFR, respectively. Covariates included in the model were age (< 45 years, 45-64 years, ≥ 65 years), gender (male, female), BMI (< 24.0 $kg{\cdot}m^{-2}$, 24.0-27.9 $kg{\cdot}m^{-2}$, ≥28 $kg{\cdot}m^{-2}$), smoking status (non-smoker, smoker, ex-smoker), drinking status (non-drinker, drinker, ex-drinker), diabetes (no, yes), hypertension (no, yes), TG (≤1.20 mmol/L, 1.21-2.00 mmol/L, ≥ 2.01 mmol/L) at baseline when they were not the strata variables.

sUA, serum uric acid; CKD, chronic kidney disease; *HR*, hazard ratio; *CI*, confidence interval

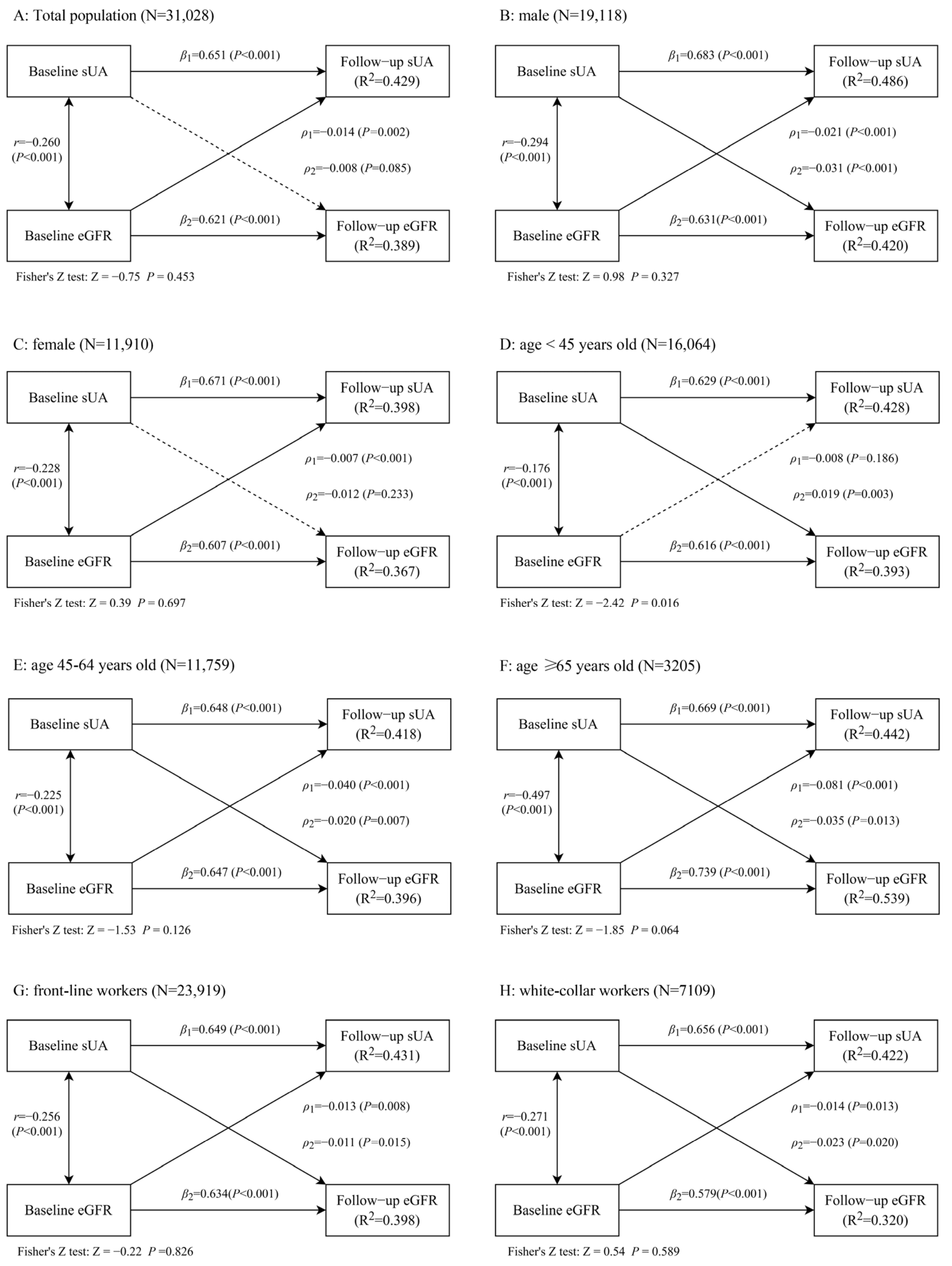

Supplementary Figure S3. Sub-analyses of the cross-lagged model of the association between sUA with eGFR, stratified by gender, age, and occupation. (eGFR was estimated by the modified Modification of Diet in Renal Disease equation).

(A: Total population, B: male, C: female, D: age < 45 years old, E: age between 45 and 64 years old, F: age ≥65 years old, G: front-line workers, H: white-collar workers).

Covariates included in the model were follow-up time, age, gender (male, female), BMI, TC, TG, diabetes (no, yes), hypertension (no, yes), smoking status (non-smoker, smoker, ex-smoker), drinking status (non-drinker, drinker, ex-drinker), education (junior high

school or below, high school, junior college, bachelor's degree or above) when they were not the strata variables, among which the multi-categorical variables smoking, drinking and education were included in the model as dummy variables. sUA, serum uric acid; eGFR, estimated glomerular filtration rate; ρ_1, cross-lagged path coefficients from baseline eGFR to follow-up sUA; ρ_2, cross-lagged path coefficients from baseline sUA to follow-up eGFR; *r* represented synchronous correlations; β_1 and β_2 represented tracking correlations; R^2, variance explained; *RMR*, root mean-square residual; *CFI*, comparative fit index.

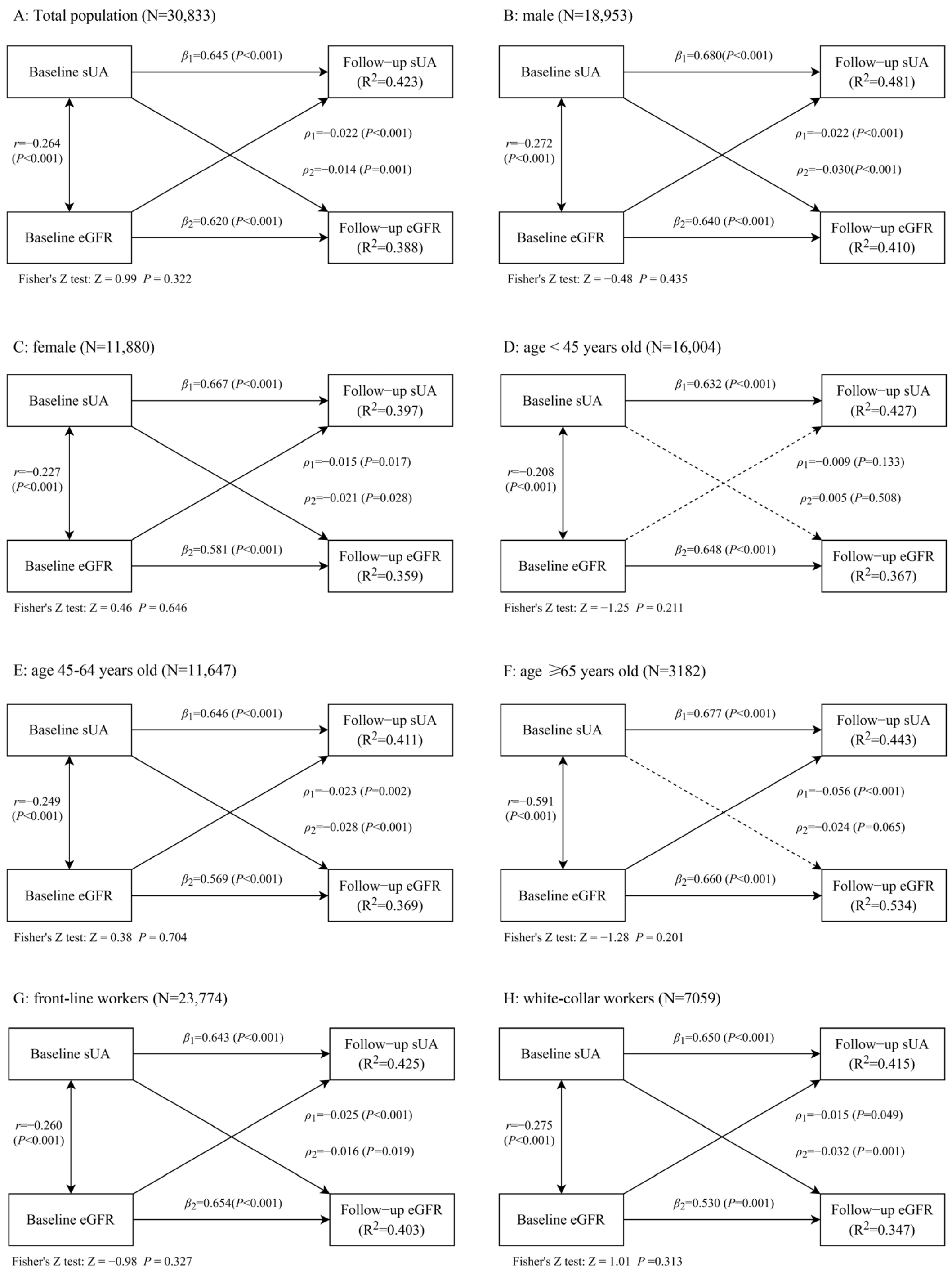

Supplementary Figure S4. Sub-analyses of the cross-lagged model of the association between sUA with eGFR, stratified by gender, age, and occupation, after excluding 195 participants with gout at baseline (sensitivity analysis).
(A: Total population, B: male, C: female, D: age < 45 years old, E: age between 45 and 64 years old, F: age ≥65 years old, G: front-line workers, H: white-collar workers).
Covariates included in the model were follow-up time, age, gender (male, female), BMI, TC, TG, diabetes (no, yes), hypertension (no, yes), smoking status (non-smoker, smoker, ex-smoker), drinking status (non-drinker, drinker, ex-drinker), education (junior high

school or below, high school, junior college, bachelor's degree or above) when they were not the strata variables, among which the multi-categorical variables smoking, drinking and education were included in the model as dummy variables. sUA, serum uric acid; eGFR, estimated glomerular filtration rate; ρ_1, cross-lagged path coefficients from baseline eGFR to follow-up sUA; ρ_2, cross-lagged path coefficients from baseline sUA to follow-up eGFR; r represented synchronous correlations; β_1 and β_2 represented tracking correlations; R^2, variance explained; *RMR*, root mean-square residual; *CFI*, comparative fit index.
